# Da Vinci single-port-assisted subcostal esophagectomy (SP SC RAMIE) – a promising SP approach

**DOI:** 10.1007/s00464-025-12383-z

**Published:** 2025-11-17

**Authors:** Vladimir J. Lozanovski, Luca Bellaio, Edin Hadzijusufovic, Evangelos Tagkalos, Franziska Renger, Olga Adamenko Meier, Suzanne S. Gisbertz, Hauke Lang, Peter P. Grimminger

**Affiliations:** 1 Department of General, Visceral and Transplantation Surgery, University Medical Center of the Johannes Gutenberg University Mainz, Langenbeckstr. 1, 55131 Mainz, Germany; 2 Department of Surgery, Amsterdam UMC Location University of Amsterdam, Amsterdam, The Netherlands; 3 Cancer Center Amsterdam, Amsterdam, The Netherlands

**Keywords:** Esophageal surgery, Minimally invasive surgery, Robotics, Subcostal, Single port, Da Vinci robot-assisted esophagectomy, Ivor Lewis esophagectomy, RAMIE

## Abstract

**Introduction:**

Single-port subcostal robotic-assisted minimally invasive Ivor Lewis esophagectomy (SP SC RAMIE) offers advantages in esophageal cancer surgery but remains unfamiliar and requires a long learning curve. This retrospective case series study evaluates its feasibility, safety, and short-term outcomes.

**Methods:**

The first 25 consecutive SP SC RAMIE procedures were analyzed. Patient demographics, neoadjuvant therapy, resection margins, lymph node yield, intraoperative parameters, postoperative recovery, complications, and mortality were assessed. Feasibility was defined by achieving R0 resection and a lymph node yield exceeding benchmark recommendations.

**Results:**

The cohort consisted primarily of male patients with adenocarcinoma of the esophagogastric junction, 72% of whom had undergone neoadjuvant therapy. R0 resection was achieved in all cases, with a mean lymph node yield of 30 (SD ± 10), confirming procedural feasibility. Mean total operative time was 324 (± 51) minutes, with an active console time of 79 (± 17) minutes. No intraoperative complications or conversions occurred. No patient received epidural or intercostal catheter analgesia and mean postoperative pain scores were very low. By postoperative day five, 92% of patients were receiving analgesia on demand. Most patients ambulated on the first postoperative day, and the median hospital stay was seven days (range 5–40). Postoperative complications included two cases of pneumonia (8%) and two anastomotic leakages (8%), all managed endoscopically with an endoluminal vacuum sponge. In-hospital, 30-day, and 90-day mortality rates were 0%.

**Conclusions:**

SP SC RAMIE is a feasible and safe esophagectomy technique for lower esophageal cancer resection, with a low complication rate and high lymph node retrieval. Rapid recovery, low pain scores, and a short hospital stay highlight its potential benefits.

**Supplementary Information:**

The online version contains supplementary material available at 10.1007/s00464-025-12383-z.

The standard curative treatment for locally advanced esophageal cancer includes multimodal therapy, consisting of chemotherapy or chemoradiotherapy and a radical esophagectomy [1, 2]. Minimally invasive esophagectomies are superior to traditional open surgery [3–6]. Furthermore, numerous randomized controlled trials have shown that robot-assisted minimally invasive esophagectomy (RAMIE) is feasible and even more suitable, as it overcomes the limitations imposed by rigid instruments and reduced freedom of movement during minimally invasive esophagectomy (MIE) [5, 7–9]. Typically, robot-assisted esophageal surgeries use either transhiatal or transthoracic approaches and are associated with significantly less blood loss, reduced postoperative pain, a lower incidence of postoperative cardiopulmonary complications, and better quality of life [10]. Moreover, RAMIE is associated with a higher yield of harvested thoracic lymph nodes and lymph nodes along the left laryngeal nerve (79.5 vs. 67.6%, *p* = 0.001), as shown in the RAMIE trial [11]. Although long-term data analysis is lacking, a lymph node yield of at least 15 resected nodes has proven to be crucial for improved overall survival [12–14]. The recently published REVATE randomized controlled trial compared RAMIE to MIE in patients with esophageal squamous cell carcinoma and aimed to prove that RAMIE is associated with a higher success rate of left recurrent laryngeal nerve lymph node dissection, with success defined as the removal of at least one lymph node without causing nerve damage lasting longer than 6 months [15]. In 203 patients from three Asian centers included in the analysis, the rate of successful left recurrent laryngeal nerve lymph node dissection was higher in the robotic group (88.3 vs. 69%, *p* < 0.001). Patients in the RAMIE group had a significantly lower incidence of left recurrent laryngeal nerve palsy compared to patients in the MIE group (20.4 vs. 34%, *p* = 0.029), and the permanent recurrent laryngeal nerve palsy rates at six months were 5.8% and 20% (*p* = 0.003). Additionally, the yield of resected mediastinal lymph nodes was higher in the robotic group (*p* = 0.035) [15].

Recent developments in robotic systems, such as the introduction of the da Vinci Single-Port (SP) Robot System by Intuitive Surgical Inc., Sunnyvale, CA, USA, allow for precise access to the esophagus through a small cervical and subcostal single-port incision [16–18]. As previously demonstrated, the subcostal incision during the da Vinci Single-Port Subcostal RAMIE (SP SC RAMIE) is used for access to the thoracic cavity to dissect the esophagus, retrieve the specimen, and perform reconstruction [18].

This study aimed primarily to report the early outcomes of our initial experience with the SP SC RAMIE procedure. The study also aimed to investigate the feasibility of this innovative approach in a larger patient cohort and to evaluate whether the SP SC RAMIE procedure is associated with reduced pain while enhancing patient mobility, thereby lowering the risks of thromboembolism and pulmonary complications like pneumonia. Moreover, the yield of retrieved lymph nodes and the rate of microscopic tumor-free margins were also assessed.

## Patients and methods

All SP SC RAMIE procedures were carried out at the University Hospital Mainz, within the Department of General, Visceral, and Transplantation Surgery, using the da Vinci SP System (Intuitive Surgical Inc., Sunnyvale, CA, USA) along with the associated instruments. This retrospective case series study was conducted in accordance with the IDEAL framework for surgical innovation and STROBE guidelines for observational research, ensuring transparent and comprehensive reporting of the study design, data collection, analysis, and interpretation [19, 20]. As a novel technique, SP SC RAMIE was introduced and evaluated in a stepwise manner consistent with Stage 2a/2b of the IDEAL recommendations, focusing on technical refinement, safety, feasibility, and initial outcomes.

All consecutive patients with distal esophageal carcinoma or AEG were eligible for SP SC RAMIE and were included in the study. Patients with tumors extending above the tracheal bifurcation or with suspicious upper mediastinal lymph nodes on preoperative computed tomography were not considered for the procedure. This was because of the limited length of the da Vinci SP instruments, which may compromise oncologic radicality and esophageal preparation above the azygos vein.

All SP SC RAMIE procedures were performed by a dedicated and consistent surgical team within a single institution, ensuring standardization of the procedure.

Data were prospectively collected and systematically documented in an institutional database. Comprehensive follow-up was performed for all patients, ensuring complete outcome assessment.

All patients provided informed consent permitting anonymous data and follow-up collection, with potential use for scientific analysis. In accordance with federal state regulations (state hospital law §36 and §37) and the independent ethics committee, no ethical approval was required for this study.

### Patient characteristics and perioperative data

We analyzed the first 25 consecutive da Vinci SP SC RAMIE procedures performed on adult patients since May 1, 2024, at our institution. Patients who underwent SP SC RAMIE were identified from a prospective institutional database, and data were extracted from both written and electronic medical records. Demographic data, including age, sex, and body mass index (BMI; kg/m^2^), were collected. Tumor location was classified according to the Japanese Classification of Esophageal Cancer [21]. Perioperative data were collected and analyzed, including diagnosis, preoperative chemoradiotherapy, tumor-node-metastasis (TNM) stage, resection margin status (R), and Charlson Comorbidity Index (CCI) [22].

Preoperative gastroscopy with endoscopic pyloric balloon dilation was performed in all patients with nonocclusive tumors.

Additional variables included the total duration of the procedure, duration of the thoracic phase, active instrument time, length of stay in the intensive or intermediate care unit, and readmission to these units. The total procedure duration was defined as the time elapsed between the abdominal skin incision and the completion of the thoracic skin suture (minutes). The duration of the thoracic phase was measured from the thoracic skin incision to the thoracic skin suture (minutes). The active console time represented the cumulative period during which the robotic instruments were engaged, reflecting the technical execution of esophageal dissection and preparation by the surgeon at the console (minutes).

Postoperative pain was assessed using the visual analog scale (VAS), and pain management strategies (peridural analgesia [PDA], paravertebral analgesia [intercostal catheter; ICC], patient-controlled analgesia [PCA], and on-demand analgesics) were prospectively recorded. Additionally, patient mobilization (postoperative day), total hospital length of stay (days), perioperative complications, intraoperative mortality, in-hospital mortality, 30-day and 90-day mortality were recorded. Perioperative complications were classified according to the Esophagectomy Complications Consensus Group (ECCG) [23].

Patients were routinely followed in the outpatient clinic every three months after discharge, with complete follow-up data available for all patients up to their most recent clinic visit before this analysis.

### Feasibility criteria

The procedure was considered feasible if an R0 resection was achieved and at least 15 lymph nodes were retrieved, in accordance with current benchmark recommendations [12–14]. The dissection and retrieval of lymph nodes were standardized by anatomical stations, with each station submitted as a separate specimen to the pathologist, as is routinely performed at our institution.

### Surgical technique

**Fig. 1:**
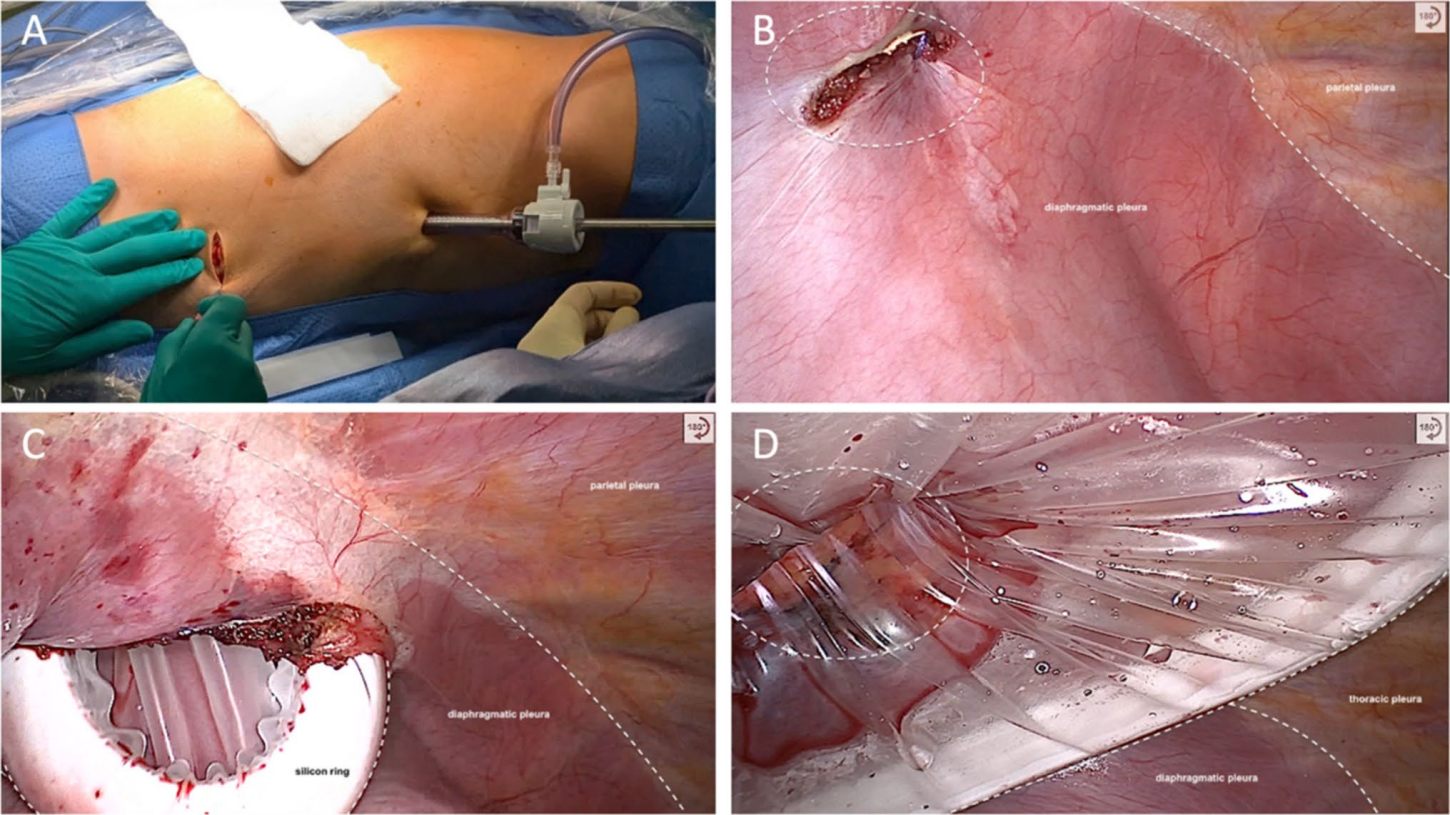
Subcostal approach to accessing the thoracic cavity. **A** Skin incision with the assistant trocar in place. **B** Intrathoracic view of the subcostal thoracotomy, showing the parietal and diaphragmatic pleura. **C** Placement of the access port. **D** SP access port in place

**Fig. 2:**
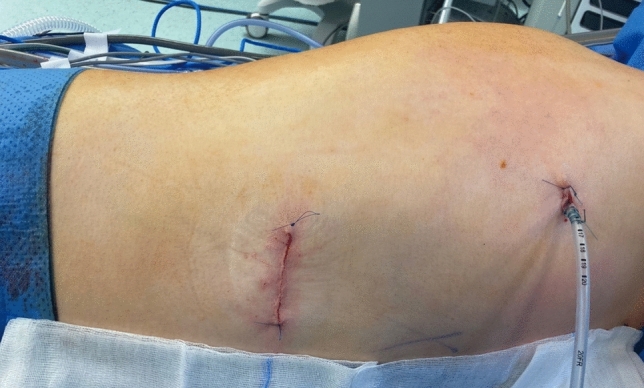
Final steps of the procedure: Skin suture of the thoracotomy wound and placement of a secured thoracic drain

This approach has been previously described in detail [18]. During the da Vinci SP subcostal phase, a small, approximately 4 cm wide subcostal incision is made at the level of the posterior axillary line, and the thoracic cavity is entered while ensuring that the diaphragm is not injured (Fig. 1). The diaphragm can be fixed to the fascia with stay sutures. A large SP access port (SP access Port Kit, Large incision (2–7 cm)) is inserted, and a pressure of 7 mmHg is applied. Once capnopneumothorax is established and the robotic system is docked, surgical instruments are positioned. The technical steps mirror those of the multi-port Ivor Lewis RAMIE procedure, although the subcostal approach results in different instrument angulation, as shown in Supplementary Video 1 and discussed in detail elsewhere [18]. The skin sutures mark the end of the procedure (Fig. 2).

### Data analysis

SPSS (Version 30.0.0) was used for all analyses. Continuous variables are presented as mean ± standard deviation (SD) or median (range), while categorical variables are expressed as percentages.

## Results

### Patient characteristics

The demographic characteristics are presented in Table 1. One patient was female, and the median age was 66 years (range: 29–89). The median BMI of the cohort was 26.7 kg/m^2^ (range: 20–41.4). Twenty patients (80%) had AEG, while five patients (20%) had squamous cell carcinoma of the middle or distal esophagus. Eighteen patients (72%) had undergone neoadjuvant treatment, either chemotherapy according to the FLOT regimen (with or without immunotherapy) or chemoradiotherapy following the CROSS regimen. According to the American Society of Anesthesiologists (ASA) Physical Status Classification System, nine patients (36%) were classified as ASA II, 15 patients (60%) as ASA III and one patient (4%) as ASA IV. The median Charlson Comorbidity Index (CCI) was 5 (range: 2–8), indicating a moderate to high burden of comorbid conditions associated with increased perioperative risk and overall mortality in the cohort. Gastroscopy with endoscopic pyloric balloon dilation was performed preoperatively in 23 patients (92%) (Table 1).

**Table 1:** Patient demographics and tumor characteristics

	N = 25
Age (years) [median (range)]	66 (29–89)
Sex [n (%)]	
Male	24 (96%)
Female	1 (4%)
BMI (kg/m^2^) [median (range)]	26.7 (20–41.4)
American Society of Anesthesiologists (ASA) Physical Status Classification System	
ASA 2	9 (36%)
ASA 3	15 (60%)
ASA 4	1 (4%)
Charlson comorbidity index (CCI) [median (range)]	5 (2–8)
Previous surgery [n (%)]	9 (36%)
Preoperative pylorus dilatation [n (%)]	23 (92%)
Tumor type and location [n (%)]	
Adenocarcinoma [n (%)]	20 (80%)
Middle esophagus	0 (0%)
Lower esophagus	2 (10%)
AEG type I	11 (55%)
AEG type II	7 (35%)
Squamous cell carcinoma [n (%)]	5 (20%)
Middle esophagus	2 (40%)
Lower esophagus	3 (60%)
Neoadjuvant treatment [n (%)]	
No treatment	7 (28%)
Chemotherapy (FLOT)	14 (56%)
Chemoradiotherapy (CROSS)	3 (12%)
Chemotherapy (FLOT) and immunotherapy (Pembrolizumab)	1 (4%)

### Operative parameters

The mean total procedure duration was 324 (SD ± 51) minutes, the mean duration of the thoracic phase was 129 (± 33) minutes, and the mean active console time during the thoracic phase was 79 (± 17) minutes (Table 2). On average, five instruments—camera in cobra position, fenestrated bipolar forceps, round tooth retractor, monopolar curved scissors, and needle holder—were used during this phase, with a mean of five instrument exchanges.

**Table 2:** Operative details, perioperative and postoperative outcomes

	N = 25
Total procedure duration (minutes) [mean (SD)]	324 (± 51)
Duration of the thoracic phase (minutes) [mean (SD)]	129 (± 33)
Active console time (minutes) [mean (SD)]	79 (± 17)
Intensive care unit stay (days) [median (range)]	0 (0–23)
Intermediate care unit stay (days) [median (range)]	1 (1–1)
Pain management	
Peridural analgesia (PDA) [n (%)]	0 (0%)
Paravertebral analgesia (ICC) [n (%)]	0 (0%)
Patient-controlled analgesia (PCA)	
Postoperative day 1 [n (%)]	11 (44%)
Postoperative day 3 [n (%)]	6 (24%)
Postoperative day 5 [n (%)]	2 (8%)
Patient mobilization (days) [median (range)]	1 (1–4)
Complications [n (%)]	6 (24%)
Pulmonary	5 (20%)
Pneumonia	2 (8%)
Pleural effusion	3 (12%)
Anastomotic leak	2 (8%)
Pulmonary embolism	1 (4%)
Clavien-Dindo complication grading system	
< IIIb	3 (12%)
≥ IIIb	3 (12%)
Hospital stay (days) [median (range)]	7 (5–40)
Intraoperative mortality [n (%)]	0 (0%)
In-hospital mortality [n (%)]	0 (0%)
30-day mortality [n (%)]	0 (0%)
90-day mortality [n (%)]	0 (0%)
Readmission to the intensive care unit	1 (4%)
Radical (R0) resection [n (%)]	25 (100%)
Number of lymph nodes harvested [mean (SD)]	30 (± 10)
Pathological TNM classification—pTNM [n (%)]	
pT0	2 (8%)
pT1a	5 (20%)
pT1b	8 (32%)
pT2	2 (8%)
pT3	8 (32%)
pT4a	0 (0%)
pN0	14 (56%)
pN1	6 (24%)
pN2	3 (12%)
pN3	2 (8%)
pM0	24 (96%)
pM1	1 (4%)

### Postoperative complications and recovery

No conversions were required during either the abdominal or thoracic phases of the procedure. No intraoperative complications occurred, and most patients recovered quickly, with a median one-day stay in the intermediate care unit. However, two patients required prolonged intensive care unit treatment: one was readmitted on postoperative day 5 due to an anastomotic leakage, while the other remained in the intermediate care unit due to respiratory distress and postoperative delirium.

In total, six (24%) patients experienced postoperative complications. Two patients developed pneumonia, which was successfully treated with antibiotics and thoracentesis on the normal ward. The very low pneumonia rate (8%) may be explained by the excellent pain management and very early patient mobilization. Two patients had an anastomotic leakage on postoperative days 4 and 5, which was successfully managed with an endoluminal vacuum sponge (Eso-SPONGE®, Braun Surgical, S.A. Spain) and antibiotics on the ward and intensive care unit, respectively. No patient developed delayed wound healing or laryngeal nerve paresis. The anastomotic leakage rate in the cohort was 8%, and the overall complication rate was 24%, with only 12% of patients experiencing complications classified as Clavien-Dindo grade IIIb or higher (Table 2). To date, apart from reflux and delayed gastric emptying in five patients (20%), which were managed with endoscopic pyloric balloon dilatation, no further complications have occurred.

### Pain management, mobilization, and readmission

Pain management was interdisciplinary and involved the anesthesiology team. None of the patients received any kind of pre- or intraoperative pain catheter management, such as PDA or paravertebral analgesia (ICC), which is routinely used with the transthoracic multi-port approach at our center (data not shown). During the first postoperative day, patients received continuous intravenous metamizole with subcutaneous piritramide on demand. From postoperative day 2 onward, they received short intravenous metamizole infusions with subcutaneous piritramide on demand, or PCA with piritramide for severe pain. Once oral intake was feasible according to the stepwise diet advancement protocol, analgesia was transitioned to oral metamizole. The mean pain intensity on the VAS on postoperative days 1, 2, and 5 was 2 (± 2). In two patients (8%) PCA was removed on postoperative day 5, whereas the majority were already receiving analgesics on demand. This facilitated very early mobilization, with most patients ambulating on the first postoperative day, and resulted in a short median hospital stay of seven days. In-hospital mortality was 0% (Table 2). However, three patients (12%) were readmitted within 30 days due to delayed gastric emptying.

### Oncologic short-term outcomes and feasibility

Radical (R0) resection was achieved in all cases. The mean number of lymph nodes harvested was 30 (± 10), exceeding current benchmark recommendations. Lymph node involvement was confirmed histopathologically in 11 (44%) patients. 30-day and 90-day mortality rates were 0% (Table 2). Overall survival was 100%.

## Discussion

This study evaluated the SP SC RAMIE procedure for the first time in Europe in an initial patient cohort, focusing on technical feasibility, surgical radicality, postoperative pain, and mobility. The results show that SP SC RAMIE is feasible, allows effective intrathoracic esophageal mobilization, and enables adequate subcarinal and radical mediastinal lymphadenectomy in selected patients. These outcomes were achieved within operative times comparable to other minimally invasive esophagectomy techniques [24–27]. An R0 resection was achieved in all patients, with a high lymph node yield likely attributable to the enhanced visualization and precision of the robotic system. These findings support SP SC RAMIE as a promising option for selected cases.

The patient cohort predominantly consisted of male individuals with AEG, with a smaller subset of patients diagnosed with squamous carcinoma of the esophagus. This reflects the typical demographic and tumor distribution in esophageal cancer [28]. A significant proportion of patients underwent neoadjuvant therapy, in accordance with current treatment protocols for esophageal cancer, highlighting the advanced stage of the disease in many cases [29–32]. Histopathological analysis confirmed the absence of lymph node involvement in 56% of cases. These results underscore the multidisciplinary care approach at our center and the technical feasibility of SP SC RAMIE.

Postoperative recovery was rapid, with nearly all patients mobilizing on the first postoperative day. The feasibility of the “single-port-less-trauma” concept was demonstrated by the absence of PDA or ICC analgesia. Patients reported low pain scores and were quickly transitioned to analgesia on demand. The subcostal incision used for access and specimen retrieval likely contributed to reduced pain and improved pulmonary function, promoting early recovery and shorter hospital stays for all patients, who were unburdened by postoperative complications. However, future randomized controlled studies should evaluate these results. Notably, the incidence of complications was very low, with only two patients developing pneumonia and two experiencing an anastomotic leak, both successfully treated endoscopically. This compares favorably with other minimally invasive and robotic-assisted esophagectomy reports [25–27]. Delayed gastric emptying, which is more related to the esophagectomy itself than to the SP SC RAMIE approach, was successfully managed in all cases. No in-hospital, 30-day, or 90-day mortality rates were observed, supporting the safety of this technique.

The da Vinci SP system has not been as widely used as multi-port systems, primarily due to its technical limitations [33]. Notably, it lacks a robotic stapler and a dedicated vessel-sealing device. Stapling in this study was performed via a separate assistant port, and the esophagogastrostomy was completed through the subcostal incision after undocking the system. This contributed to the difference between console and thoracic phase durations. The development of integrated stapling tools could reduce operative time. The technical steps of the SP SC RAMIE procedure are the same as those of the multi-port Ivor Lewis RAMIE. However, the limited length of the da Vinci SP instruments makes esophageal preparation and lymphadenectomy above the azygos vein difficult [18, 34]. To avoid compromising oncologic radicality, the SP SC RAMIE procedure was mainly performed in patients with distal esophageal cancer or AEG. Nevertheless, the da Vinci SP system helps avoid arm collisions and allows precise instrument control in confined surgical spaces [17].

There are several limitations to consider. The retrospective single-center case series study design and small sample size limit generalizability and make it susceptible to bias, despite efforts to minimize it. This limitation can be explained by the very limited national and international experience with the SP SC RAMIE procedure and restricted familiarity with the da Vinci SP system. Although the core principles of oncologic esophagectomy remain unchanged, the unique instrument angulation and the SP platform demand specific skills and a structured learning curve. This also explains why no comparison was made to the da Vinci Xi RAMIE procedure, for which the learning curve was surpassed after 22 cases at our institution [35]. Furthermore, wider implementation may be delayed by the lack of standardized training and initial unfamiliarity, which can impact outcomes. These challenges could be mitigated through institutional support, interdepartmental coordination, case observation and proctoring—areas where high-volume centers can play a crucial role. Finally, the effect of SP SC RAMIE on overall survival and recurrence remains unknown, as this platform is newly introduced.

The outcomes reported here may be comparable or even superior to established multi-port RAMIE studies, which have demonstrated reduced complications and improved lymph node yields over video-assisted laparoscopic–thoracoscopic minimally invasive techniques [11, 15]. This study aligns with those observations, showing low postoperative pain and potentially shorter hospital stays, and suggests incremental benefits worth further investigation. The median hospital stay in this study was seven days, which compares favorably with the findings of Yang et al. (MIE 13 days, RAMIE 10 days) and Chao et al. (robot-assisted esophagectomy 10 days, video-assisted esophagectomy 11 days) [11, 15]. Its structured methodology supports responsible innovation and lays the groundwork for future multicenter comparisons, in line with IDEAL recommendations. However, further research—particularly multicenter trials following the IDEAL framework—is needed to validate these early findings.

## Conclusion

SP SC RAMIE demonstrates favorable short-term outcomes, including low complication rates, rapid postoperative recovery, and high lymph node yield. Effective pain control could be achieved without epidural or paravertebral analgesia. The findings support the feasibility, safety, and technical adequacy of the da Vinci SP SC RAMIE approach for resecting AEG and cancers of the middle and lower esophagus. However, further studies are needed to confirm these findings, define long-term benefits, and evaluate oncologic outcomes. Moreover, technical enhancements, such as integrated vessel-sealing devices and robotic staplers may help streamline the procedure and support broader adoption.

## Supplementary Information

Below is the link to the electronic supplementary material.

Supplementary file1 (MP4 545520 KB)
